# 19.1 FROM CLINICAL TRIAL TO THE CLINIC: OPTIMIZING COGNITIVE ADAPTATION TRAINING FOR CASE MANAGEMENT TEAMS

**DOI:** 10.1093/schbul/sby014.075

**Published:** 2018-04-01

**Authors:** Sean Kidd, Yarissa Herman, Gursharan Virdee, Chris Bowie, Dawn Velligan, Natalie Maples

**Affiliations:** 1 University of Toronto; 2 Toronto Centre for Addiction and Mental Health; 3 Queen’s University; 4 University of Texas Health Science Center at San Antonio, School of Medicine

## Abstract

**Background:**

Cognitive Adaptation Training (CAT) has consistently demonstrated effectiveness in enhancing community functioning in clinical trials of its 9-month application by a specialist. This is a compelling development in the field as clinicians struggle to support gains in independent functioning among patients with schizophrenia. However, outreach interventions delivered by specialists are difficult to support in many contexts where investment in mental health care is insufficient for population needs. This presentation will describe research and implementation efforts that support the delivery of CAT in routine clinical practice.

**Methods:**

This program of work began with a feasibility study of a modified version of CAT. CAT was modified to decrease the duration of specialist-delivered CAT to 4 months, with the intervention subsequently supported by the individual’s case manager who received rudimentary training and could consult specialists. Twenty-three people with schizophrenia participated in this study of symptom and functional outcomes, evaluating improvements after 4 months of CAT specialist intervention and after an additional 5 months of case manager support. Also described briefly will be (i) preliminary findings from a superiority randomized controlled trial of modified CAT in an early intervention population comparing CAT (n=25) with Action Based Cognitive Remediation (n=23) and (ii) efforts to build out CAT implementation in a tertiary facility enabled through the above clinical trial resources.

**Results:**

Analysis of feasibility study findings revealed significant improvements in adaptive functioning, psychiatric symptomatology, and goal attainment that were maintained throughout case management follow-up. Effect sizes for the specialist delivered period ranged from .33 (negative symptoms) to 2.01 (goal attainment scaling) with a modest decline in the follow-up period with community functioning remaining at ES=.66. Improvement in the large effect size range was also observed in community functioning in the trial of modified CAT in early intervention. In this period over 70 allied health clinicians were intensively training in CAT locally and regionally and a community of practice was established. These impacts were further extended through the development of an open-access CAT guide for families that can be used independently or with clinician support.

**Discussion:**

This study supports a model for extending the accessibility of CAT in settings that might not otherwise sustain the intervention as it was originally designed. Functional impacts similar to the original clinical trials were observed in a briefer period of specialist delivered CAT and show the promise of being largely sustained over an indefinite period by rudimentary-trained case managers in a consultation model. This observation would appear to apply to both early intervention and general schizophrenia populations. Additionally, this program of work has demonstrated how research-practice synergies can foster implementation that can be sustained after initial research investments.

